# Knowledge and Behavioral Habits to Reduce Mycotoxin Dietary Exposure at Household Level in a Cohort of German University Students

**DOI:** 10.3390/toxins13110760

**Published:** 2021-10-26

**Authors:** Katherine Muñoz, Mara Wagner, Florian Pauli, Juliane Christ, Gerhard Reese

**Affiliations:** 1 Group of Organic and Ecological Chemistry, Institute for Environmental Sciences Landau, University of Koblenz-Landau, 76829 Landau, Germany; 2 Group of Environmental Psychology, Department of Social, Environmental and Economic Psychology, University of Koblenz-Landau, 76829 Landau, Germany; mara@wagner-sb.de (M.W.); paul7634@uni-landau.de (F.P.); juliane_christ@gmx.net (J.C.); reese@uni-landau.de (G.R.)

**Keywords:** mycotoxin exposure, knowledge, risk perception, dumpster diving, food sharing

## Abstract

Mycotoxins pose a health concern for humans. Therefore, strategies at pre- and post-harvest and maximum levels for food have been implemented, aimed to minimize the risk of dietary exposure. Yet, consumers’ dietary habits and life style play a substantial role in overall exposure. The aim of this study was to investigate knowledge of mycotoxins and accordance to behavioral practices or habits that may affect the risk of mycotoxin dietary exposure at the household level or when food commodities are obtained from non-regulated trade markets. For this purpose, an online survey was applied to a university student cohort (*n* = 186). The survey consisted of 23 questions grouped in five categories: Socio-demographic and income data, general life style and habits, knowledge about mycotoxins, compliance with the “17 golden rules to prevent mycotoxin contamination” of the German Federal Institute for Risk Assessment (BfR), and measures towards reducing health risks. We paid particular attention to knowledge and compliance of a group acquiring food items in markets outside regulation and surveillance, namely, adherents of food movements such as food sharing or dumpster diving. The results of our study indicate a generally rather low level of knowledge about mycotoxins in the investigated cohort, as well as a weak perception of their associated risks compared to similar studies; around half of the cohort was unfamiliar with the term “mycotoxin” and the health risks of mycotoxins were considered comparable to those of pesticides, heavy metals, microplastics and food additives. We observed, in general, a relatively high degree of compliance with the proposed golden rules. The rules with the highest compliance related to deteriorated foods with visible signs of fungal infestation, probably because these are already considered as food waste. Rules that were less followed included those that require a specific knowledge of food storage and early fungal contamination stages, namely preventive measures related to storage of bread. Adherents of food movements did not differ significantly with the control group in terms of knowledge, risk perception and compliance with the 17 golden rules. This may be due to the homogeneity of the cohort in terms of demography, age and educational level. However, significant low compliance in the food movements group was observed with the rules “Buy fruit and vegetables that are as intact as possible, i.e., without injuries and bruises” and “Rotten fruit should neither be eaten nor further processed into compote or jam”, possibly because of ideological convictions around reducing food waste. In conclusion, mycotoxin prevention strategies should not end at the retail level; in particular, clarification and information regarding health risk from mycotoxins are suggested in order to reduce the risk of exposure in private households or in informal trade markets. The results of this study should, however, be interpreted with caution due to the specific characteristics of the cohort in terms of age and educational level and the disparity in size between the control and the food movement group. This study is a starting point for evaluating and understanding the consumer perspective on mycotoxins.

## 1. Introduction

Mycotoxins are secondary metabolites synthesized by filamentous fungi, chemically diverse so that they exert a broad spectrum of toxic effects to animals and humans [1]. Aflatoxins, ochratoxins and fusarium toxins belong to the most investigated toxins because of their toxicities (Table 1) and their wide occurrence in environmental as well in human samples. Mycotoxins are primarily produced after fungal infestation in crops and food and feed commodities. According to recent estimates, far more than 25% of global food crop production is contaminated with mycotoxins [2]. Humans can be exposed to a diversity of mycotoxins [3], the main route of exposure being the intake of contaminated foods [4,5]. Dietary exposure is, however, determined by alimentary habits and lifestyle [6]. In the same way, the toxic effects that mycotoxins may exert are directly linked to the magnitude of the dose and to the health status of the population. Shephard [7] pointed out the situation in African countries with environmentally relevant mycotoxin concentrations, as well as the instability of health systems in the respective countries, as exemplified by aflatoxin B1 and liver cancer. Monitoring studies in food commodities as well as human monitoring show that continuous exposure to mycotoxins is possible [8,9]. In addition, high occurrence and levels of mycotoxins in the diet may pose a serious health concern [10] in light of associations between relative high incidences of *Fusarium* toxins and esophageal cancer [11], aflatoxin B1 and hepatocellular carcinoma [12,13] or gallbladder cancer [14,15]. Further, mouldy food or the occurrence of aflatoxins in food items is considered a key dietary factor associated with increased cancer risk in the population [16].

In order to protect consumers and to minimize exposure to mycotoxins via food consumption, maximum limits for mycotoxins have been set in food commodities [17,18] and tolerable daily intake values (TDI) have been proposed [19]. Strategies for prevention, control, reduction or mitigation of mycotoxin-contaminated commodities have also been implemented at pre- and post-harvest stages along the food chain [20,21,22], intended to reduce contaminant levels in raw and processed food. The common goal of these strategies is to prevent or hinder fungal growth and mycotoxin production by influencing the environmental conditions. Accordingly, storage after harvest has been identified as a critical stage when temperature, humidity, aeration or light conditions may shape fungal proliferation and, subsequently, mycotoxin production [23,24]. Furthermore, preventative strategies and sanitary measures that guarantee the quality of food at all stages of the food chain until commercialization at retail market do exist at the national and international levels, combined with continuous surveillance monitoring of commodities performed by reference laboratories [18,25,26]. Inspections are primarily carried out to check the compliance with hygiene regulations or the traceability, composition and labelling of the products. Monitoring programmes are conducted yearly in order to measure and evaluate levels of substances that are undesirable from a health point of view [27]. These include the monitoring of regulated mycotoxins [17] in foods of animal and plant origin, through the food chain and above all at retail level. The results of food monitoring studies disclose low occurrence and levels of mycotoxins in products at retail across European countries [28,29,30].

The above-mentioned measures aimed to reduce the dietary exposure may, however, be insufficient to protect consumers. On the one hand, dietary habits and food preferences play a significant role in exposure to contaminants [31]. On the other hand, stages after retail are not considered in monitoring studies, and mycotoxin production may also occur at the household level or when commodities are acquired in markets beyond regulation. Consumers can contribute to reducing their risk of exposure via suitable conditions for storage and processing of food. A prerequisite, however, is a certain degree of knowledge on what mycotoxins are, their risks, their sources, and prevention strategies [32]. In 2018, the German Federal Institute for Risk Assessment (BfR) published a consumer information document entitled “Mould toxins in food—How to protect yourself” [33]. The document consists of eight pages with introductory information about what mycotoxins are, their occurrence in foods, and information about symptomatology in case of acute exposure. At the end, the document includes a list of 17 “golden rules” that suggest measures to reduce domestic mycotoxin contamination in foods (Table 2). These rules advise optimal conditions for storage, processing and use of food commodities, similar to the USDA recommendations for consumers “Moulds on food: Are they dangerous?” [34]. Although most of the rules convey general knowledge and belong in part to everyday life situations, a lack of information regarding mycotoxin production and health risk [35] together with individual convictions and ideologies regarding food waste [36] may pose an additional risk in terms of a transitory elevated exposure.

Actions to reduce food waste resulted in the founding of food movements such as food sharing and dumpster diving, with an increasing appeal and adoption among young population [37]. These movements are united by the motivation to raise awareness of food waste and by tendencies to anti-consumption [38]. Food sharing consists of the exchange of food, mostly of no longer saleable groceries from supermarkets, meals or leftovers from commercial and private sources through non-traditional or informal channels such as social networks and online platforms [39,40]. Participants in food sharing collect food from a variety of food providers before it is thrown away or enters a ‘waste’ state and share it for free regardless of the social status [41]. Dumpster diving involves the collection of no longer saleable but usually still edible food from grocery store food waste containers, for example from supermarkets, for domestic consumption [38]. Other than food sharing, participation in dumpster diving also represents a survival strategy for people living in poverty who experience food insecurity [42]. In Germany, the legal situation of dumpster diving is fairly complex, since the ownership of the items remains with the retailer until the dumpster is moved to where the garbage collection service will pick it up [43]. There is no reliable data on health concerns associated with food sharing or dumpster diving. Further, this practice goes beyond the risk associated to food poisoning [42]. However, the informality and the goodwill nature of food sharing may pose risks, especially health and safety risks, since the distribution of food outside of the official regulatory system evades government health, hygiene and safety regulations [40,44]. 

Consumer knowledge on preventative methods to reduce food safety threats will lead to changes in food consumption habits and to reduced concerns [45]. However, less attention has been paid to consumers’ views and knowledge of mycotoxins as biohazards in food items and the health risks associated with their exposure [1], in particular outside of the regulatory system at household level [46,47]. Ortiz, et al. [48] suggested that capacity building and public awareness are the main tools to “fight mycotoxins” worldwide. Furthermore, communication strategies have been developed and suggested aimed at improving the knowledge of the population about mycotoxins and the health risks associated with them [49]. The present study addresses the social aspects of the subject and aims to evaluate the level of knowledge and awareness on mycotoxins, as well as prevention strategies or habits such as compliance with the 17 “golden rules” among young people in a university cohort, including supporters of the food sharing and dumpster diving communities. We hypothesized that: (i) Mycotoxins are known, but the health hazards and risks are not properly estimated; (ii) Not all of the suggested rules for reducing mycotoxin contamination (17 golden rules) have the same degree of compliance, since this is strongly related to daily life habits, food preferences and behavior; (iii) Participants in food movements such as food sharing and dumpster diving may have an additional risk of mycotoxin exposure via food consumption, since the practicability of these movements might not agree with the suggested rules.

## 2. Results

### 2.1. Knowledge on Mycotoxins and Health Risk Perception

In the present study, 54% of the participants stated that they were familiar with mould toxins and 48% of all respondents answered the question “Have you ever heard the term mycotoxins?” with Yes. Half of the participants (50%) rated the health risks of mycotoxins in general to be “rather risky” to “extremely risky”. Similarly, 55% of the participants indicated that they were “concerned” or “very concerned” about the occurrence of mycotoxins in food. After an intervention text, 64% of them answered that they were already aware of the health hazards posed by mould toxins in foods, indicating also that mycotoxins may pose a major risk for young children (83%). To assess risk perception, participants were asked to compare the health risk posed by mycotoxins with other risks in food, namely pesticides, heavy metals, microplastics and food additives. The majority of the participants classified the risk of mycotoxins in food to be at least equally high as the risk from the above-mentioned xenobiotics (Table 3). However, the risk of mycotoxins compared to food additives was considered to be “somewhat worse” to “much worse” by the majority of the participants (61%). In this cohort, 70% of the participants answered that as consumers, they have the choice to protect themselves from mycotoxin exposure. In particular, 60% of them rated their influence on the prevention of mycotoxins in their own food to be rather high to very high. Furthermore, 56% of the respondents considered that the absence of mycotoxins in food may strongly protect their health. 

### 2.2. Compliance with the Golden Rules

**Figure 1:**
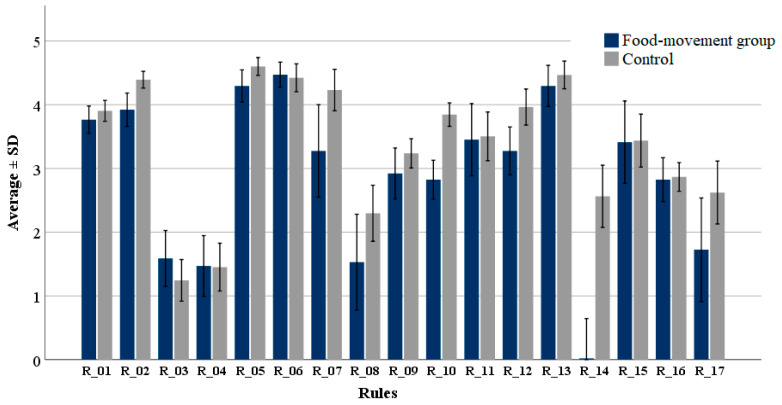
Degree of compliance with the 17 golden rules suggested by the BfR. Respondents were divided in two groups: participants in food movements (food-movement group) and non-participants (control). The scale in the Y axis represents the degree of compliance: 1: “I never follow”, 2: “I usually follow”, 3: “Partly/partly”, 4: “I follow most of the time”, 5: “I always follow”. The description of the single rules is presented in Table 2.

The observed compliance with the 17 golden rules (Table 2) was relatively high (3.2 ± 1.7) in the cohort, in the scale from 1 (I never follow) to 5 (I always follow); however, the degree of adherence among participants varied between the individual rules (Figure 1). Preventative measures were estimated with an average value of 3.1 ± 1.3, similar to the value observed for measures of how to act in case of mouldy food (3.3 ± 2.1). The highest compliance was observed with rules 2 (average 4.3 ± 0.8), 5 (average 4.5 ± 0.8), 6 (average 4.4 ± 1.1) and 13 (4.4 ± 1.2); the relative number of participants answering “I always follow” was 44%, 67%, 68% and 73% respectively. Rule 2 relates to optimal condition for storage of food commodities while rules 5, 6, and 13 refer to an evident and visible mould infestation. The lowest compliance was observed with rules 3 (average 1.3 ± 1.8) and 4 (average 1.5 ± 2.1), which correspond to preventive measures related to bread. Low compliance was also observed with Rule 8, related to cheese, with an average of 2.1 ± 2.6. Adherence to the other rules ranged between 2 and below 4; for example, Rule 16, related to storage of spices (2.9 ± 1.3), with only 31% of the participants answering “I follow most of the time” or “I always follow”.

### 2.3. Knowledge, Health Risk Perception and Degree of Compliance with the Golden Rules in Participants Involved in Food Movements 

In total, 51 participants (27%) confirmed their involvement in either food sharing and/or dumpster diving. Regarding food and health risk perceptions, 61% of the food-movement group fully agreed that for them, food is an important topic, versus 49% of the group with no participation (control). Only 17% asserted that they are very well informed about food issues, similar to the control condition. The food-movement group agreed to a lesser extent with attitudes towards health risk prevention compared to the control group, the difference being significant for the presumptions “I think a lot about health risks and try to prevent them” (*p* = 0.019) and “I try to protect myself from health risks I hear about” (*p* = 0.020). The knowledge on “mould toxins” was slightly lower in the food-movement group: 49% vs. 56% in the control; however, this difference was not statistically significant. No differences between the groups were observed with the term “mycotoxins”. Both groups similarly answered to the general health risk associated to mycotoxins and by comparing the risk of mycotoxins with that of pesticides, heavy metals, microplastic or food additives.

On average, the food-movement group showed lower compliance (−14%) with the golden rules compared to the control (Figure 1). Significant differences between both groups were observed with rules 2 (*p* < 0.01), 5 (*p* = 0.015), 7 (*p* = 0.046), 10 (*p* < 0.01), 12 (*p* < 0.01) and 14 (*p* < 0.01). The largest differences were, however, observed with Rule 7, “Affected milk and milk products must no longer be consumed” (−21%), Rule 10, “Buy fruit and vegetables that are as intact as possible, i.e., without injuries and bruises” (−26%), and Rule 12, “Rotten fruit should neither be eaten nor further processed into compote or jam” (−18%). Rules 8 (−33%) and 14 (−90%) resulted in large differences, probably attributable to the food habits (vegetarian or vegan) in the risk-group.

## 3. Discussion

### 3.1. Mycotoxin Awareness and Risk Perception

The knowledge of mycotoxins among participants in this study (48%) is rated as low compared to the Consumer Monitor study published by the BfR [50], in which 82% of the participants had already heard of mycotoxins in food. However, the health risk perception in the present study was found to be higher compared to that study. In a study in Belgium [51], approximately 70% of the participants answered that mycotoxins could lead to human or animal toxicity. In general, there is a lack of knowledge by consumers regarding mycotoxins as biohazards. This is evident in the study of Koch, et al. [52], where 1001 participants in Germany were interviewed on the topic of “contaminants in food” by means of computer-assisted telephone interviews. Only three participants spontaneously listed “mould toxins” as example of undesirable substances in foods. Differences in levels of knowledge and awareness between our study group and previously published studies can be attributed to the cohorts covered in the surveys: consumer food safety knowledge and practices vary considerable across demographic categories and possibly across of socioeconomic, educational and cultural levels [53]. In the present study, the cohort exclusively consisted of university students, while other studies included a broader and more diverse representation of the population [51,52]. When comparing mycotoxins with pesticides, microplastics, heavy metals or food additives, almost no differences in terms of risk perception were observed in our student cohort (Table 3). Almost half of the respondents rated pesticides to be “equally bad to mycotoxins”. This suggests that there is a low degree of knowledge and risk perceived for mycotoxins in food. The xenobiotic categories used for comparison, namely pesticides, microplastics, etc., differ in terms of occurrence and concentration in food commodities and related toxic effects to humans. Comparing the perceived risks of mycotoxins to those of pesticides, the opinion of the surveyed participants contradicts the expert opinions: experts consider the risk to humans from mycotoxins to be more serious than the risk from appropriately used pesticides [54]. The quote from Kuiper-Goodman [55] sums up in a proper manner the expert opinion: “in terms of exposure and severity of chronic diseases, especially cancer, mycotoxins currently appear to pose a higher risk than anthropogenic contaminants, pesticides (when used as directed) and food additives”. The knowledge gap between expert and lay persons is clearly visible in our study, since certain mycotoxins have a higher health risk of causing diseases such as estrogenic or carcinogenic effects (Table 1) as a result of chronic exposure [12,13,14,15] than, for example, pesticides or food additives [16,56,57]. We observed a willingness to adapt dietary or lifestyles and food preferences in order to reduce mycotoxin dietary exposure. The way forward in order to reduce exposure is to increase awareness by consumers, policymakers and businesses of potential mycotoxin dangers and by the global adoption of principles and practices designed to reduce mycotoxin exposure [32]. Beyond this, risk communication and intervention strategies are also necessary to reduce the exposure risk at the consumer level [49,58,59].

### 3.2. Golden Rules and Consumer Protection Behaviour 

In general, the results of this study showed good compliance with the golden rules proposed by the BfR. The rules with the highest acceptance were these related to indications for food items with visible mould spoilage. Visible pests, disease, spoilage, contamination and natural drying out of food are considered as examples of food waste across the food supply chain [60]. In the study of Gaiani, et al. [61] on consumers’ attitude to food waste in Italy, mouldy food was identified as waste by more than 40% of the participants (*n* = 3087). Similarly, the study of Jörissen, et al. [62] showed that among German participants, 78% of them considered mouldy food as a reason food is wasted. Rules 3, “Clean bread boxes and similar items once a week and rinse with vinegar and water to prevent mould growth” and 4, “Remove bread crumbs from bread boxes as they favour mould growth” were the rules with the lowest compliance. These rules are preventive measures to avoid fungal spoilage and mycotoxin contamination in bread under domestic conditions. The proposed interpretation for the low degree of compliance is that consumers subjectively do not necessarily consider food with mould infestation in the early stages as food waste. Products in such as conditions seem to be further processed or consumed without any health concern [63]. Bread stored under household conditions may evidence quantifiable fungal growth (e.g., day two) even before it is perceived visually [64]. In these studies, the authors did not find positive samples for the mycotoxins ochratoxin A and fumonisins. However, bread without signs of fungal spoilage can be contaminated with mycotoxins [65], in some cases exceeding the maximum limits set for the mycotoxin ochratoxin A in processed cereal products (3.0 ng g^−1^) or in foods intended for infants and young children (0.5 ng g^−1^) [17]. The study by Legan [66] showed that the number of days without visible mould growth after inoculation in bread was on average 2.9, 3.4 and 3.8 days, respectively, for the mycotoxigenic moulds *Cladosporium sphaerospermum*, *Penicillium notatum* and *Aspergillus niger*. In bread inoculated with *P. expansum*, the mycotoxin ochratoxin A was detected up to 3.25 ng g^−1^ after only seven days’ incubation [67], independent of pH and water activity levels. Ochratoxin A is a mycotoxin produced mainly in storage stages and the production of which is mainly driven by factors such as temperature, water activity and light conditions [23,24]. These studies support the recommendations proposed by the BfR (rules 3 and 4).

In the case of rules 8 (cheese), 9 (flour) and 16 (spices), we consider the low compliance in the cohort to result from a lack of specific knowledge on mould growth stages in food commodities. It is known that cheese-contaminating mould species may produce diverse mycotoxins [68,69]. The fungal metabolites may diffuse up to 2 cm into the inner core of the hard cheese [70]. Coton, et al. [71] suggest as a rule of thumb that as long as only white mycelium has developed on the cheese surface, trimming can be acceptable, whereas blue mould colour (due to fungal sporulation) is associated with the accumulation of significant amounts of mycotoxins, and the product should be discarded. In wheat flour, the first appearance of fungal contamination occurred on the ninth day, under conditions of high humidity (75% RH) and a temperature of 21 °C, which coincide with the beginning of the caking process (formation of lumps) [72]. However, the skills necessary to decide between trimming and discarding or whether shaking is necessary may not be present among lay persons. 

### 3.3. Food Movements and the Risk of Mycotoxin Exposure via Food Consumption

Overall, no significant differences were observed between the food-movement and control groups when evaluating their knowledge of mycotoxins and perception of associated risks. This can be attributed to the similar educational and demographic background among participants, which are known determinants of food behaviour and attitudes [40,73]. However, the food-movement group had a slightly lower level of agreement with the 17 golden rules (−14%) than the control group. The largest differences between both groups were observed in case of deteriorated milk products (Rule 7) and fruits and vegetables with signs of injuries, bruises or visible rot (rules 10 and 12), with a lower compliance in the food movement group. Fruits with injuries and bruises might not necessarily be considered as waste on the basis of a consumer’s personal experiences with foods that predetermine aesthetic standards [74]. A health concern is, however, the mould rot of apples and pears caused by *P. expansum*, a patulin-producing species [75]. Patulin is a mycotoxin frequently detected in fruits and fruit products. and remains stable after processing. Beretta, et al. [76] showed that in rotten apples the amount of patulin was extraordinarily high in the rotten area, but the mycotoxin was also spread to the part unaffected by the mould. In the case of tomatoes, the mycotoxin can even penetrate the whole fruit [77], so that a discard of the affected part and further processing is not advisable. In line with our hypothesis, members of the food movements, who are highly motivated to reduce food waste, are reluctant to throw away mouldy food, as this contradicts their values. Rombach and Bitsch [38] evidenced that the motivations behind food sharing among German population are mostly ideological or identity-establishing reasons: to reduce food waste, to act against overconsumption, and to promote the value of food and food commensality. Similar results have been reported in other studies [41,78]. A concrete example of the contradictions between the BfR rules and the principles of food sharing is the handling of mouldy fruits: according to BfR “Rotten fruit should neither be eaten nor processed into compote, fruit juice or jam.”, while the hygiene guideline from “foodsharing” [79] reads “mould is less critical for groceries with a harder consistency (such as apples, carrots, etc.), the putrefaction area can be cut off generously” and the rest can be eaten. This contrasts with current scientific evidence showing that certain mycotoxins can spread to the part unaffected by the mould, for example in apples [76]. Therefore, food movement members may unknowingly expose themselves through their behaviour to a temporarily elevated mycotoxin risk, while their knowledge on mycotoxins and their associated risks is similar to the control group. 

Morrow [40] summarized the different exigencies for food safety, traceability and quality criteria for foods, with clear conflicts between regulation and legislation by the Berlin Food Safety Authority and foodsharing.de. An example is the quality criteria set by foodsharing.de: “Do not share anything you wouldn’t eat”. Knowledge of mycotoxins and preventative behavioural measures should be considered part of an integrated risk assessment beyond the retail level with a focus on potential risk groups, namely consumers acquiring products at informal markets escaping regulation and surveillance. Moreover, food movements have increasing participation among young women. Therefore, it should be also considered that, through breastfeeding, a transfer of certain mycotoxins via milk can occur [80], posing a risk to sensitive groups such as infants in early life [81,82]. For this reason, both ourselves and others [32,58,59,83,84] advise improved risk communication regarding mycotoxins as a general key strategy to reduce exposure at the household level. Further, it is suggested that the risk in consumers who participate in informal food trade markets should be assessed.

## 4. Conclusions

The results of the present study indicate a generally low level of knowledge about mycotoxins, as well as weak perception of their risks in comparison to previous studies. The risk of mycotoxins was considered similar as those of pesticides, heavy metals, microplastics and food additives. This contradicts the views of the surveyed participants and the evidence from expert opinions. The results of this study confirmed the hypothesis (i), namely, that mycotoxins are known, however, the health hazard and risk are not properly estimated by the participants in this cohort. Overall, we observed a high degree of compliance with the rules proposed by the BfR aimed at reducing exposure at the household. The rules with the highest acceptance were those in which an evident mould infestation is observed, so that food commodities are regarded as food waste and not further processed or consumed. Low compliance was observed with those rules related to prevention measures and those where specific knowledge is required to take a decision on further processing. Consequently, hypothesis (ii) could be confirmed as well: not all of the suggested rules to reduce mycotoxin exposure had the same degree of compliance in the cohort. Intentions to reduce food waste have resulted in an increasing motivation of young people to participate in food movements, namely food sharing and dumpster diving. We postulate that, besides the goodwill nature of the food movement initiatives, regular participation may pose an additional risk of exposure to mycotoxins, since these distribution channels are outside of the food regulation, control and surveillance system. Thus hypothesis (iii), “Participants in food movements such as food sharing and dumpster diving may be exposed to additional risk of mycotoxin exposure via food consumption, since the practicability of these movements might not agree with the suggested rules” could only be confirmed in five of the 17 rules. In particular, those rules related to fruits and vegetables with signs of injuries, bruises or visible rot. In conclusion, mycotoxin prevention strategies should not end at the retail level, but should be further implemented in private households and in informal trade markets in order to achieve a sustainable reduction of dietary mycotoxin exposure. Above all, risk communication strategies are advisable in order to increase knowledge and for better risk perception. The results of this study should, however, be interpreted with precaution due to the specific characteristics of the cohort in terms of age and educational level and the disparity in size between the control and the food movement group. Nonetheless, this case study represents a starting point for evaluating and understanding the consumer perspective on mycotoxins, the health risks caused by them, and prevention measures in households. With this study, we pointed out the need for an interdisciplinary approach by investigating mycotoxin dietary exposure in households. Combined efforts should consider the behavioral aspects as well as suitable monitoring studies in order to assess the relevance and efficacy of preventative measures to reduce mycotoxin exposure.

## 5. Materials and Methods

### 5.1. Cohort

The cohort consisted of 186 university students at bachelor or master level, 79% indicated to be female, 21% male. Age ranged between 18 and older than 49 years, 83% of them aged between 20–29 years. Participants showed a wide distribution in the budget destined for food items in a month: 32% spent between EUR 100 and 200, 40% between EUR 50–100 and 13% between EUR 200–300. Only 9% of the participants had a budget below EUR 50 per month. The income level distribution was in 24% of the participants below EUR 250 month, in 31% between EUR 250–499 and in 26% from EUR 500–999. There was a clear trend in the distribution of diet patterns: 40% of the participants were omnivore, 43% vegetarian and 13% vegan. In total, 61% indicated a diet based on organic products. In this study, 51 students (27%) confirmed participation in a food movement, the majority food sharing; eight students participated exclusively in dumpster diving. The frequency of participation in these activities was not evaluated in this survey. Participants in food movements (food-movement group) were compared to the control group (no participation in food movements) that consisted of students with no participation in food sharing nor in dumpster diving. Sources of information were also asked about; 39% of the participants answered that they used public online information pages and institutes’ websites, e.g., the Federal Institute for Risk Assessment, to get informed about risks associated with food, 20% through social media, and 20% via friends or acquaintances. 

### 5.2. Questionnaire 

The objectives of this study were: (1) To evaluate the knowledge and awareness of mycotoxins; (2) To investigate adherence of university students to “rules” to prevent mycotoxin dietary exposure at household level; (3) To compare knowledge, awareness and adherence in two cohorts including participants in food movements. For this purpose, an online questionnaire was developed with the survey software on the website soscisurvey.de. The questionnaire consisted of 23 items (Supplementary Material Table S1) divided in five categories: Socio-demographic and income data (items 1–8), life style and food movements (items 9–12), general knowledge about mycotoxins (items 13–15), compliance with the 17 golden rules suggested by the BfR (item 16) and behaviour towards health risks derived from mycotoxins and other xenobiotics (items 17–23). Prior to question 16 a short text with information about mycotoxins was provided; the text was obtained from the document “Mould toxins in food—How you can protect yourself” published by the BfR. The questionnaire was available between December 2019 and January 2020. The channels of distribution were the university’s survey distribution list (University of Koblenz-Landau), private groups in messengers such as Whatsapp and Telegram, social media, namely Facebook and Instagram, and printed flyers with QR codes distributed within the university, on the street, and in private surroundings. The questions were in German and included selection and assessment tasks containing both single and multiple answers. To assess compliance with the golden rules, a metric scale was used. The 17 golden rules were classified in two categories: (a) preventative measurements for food with no signs of mould infestation (rules 1, 2, 3, 4, 6, 9, 10 and 16); and (b) measures on how to proceed in case of a visible mould infestation in food (rules 5, 7, 8, 11, 12, 13, 14, 15 and 17). The quality of the questionnaire in terms of understandability, correctness and time was pretested with *N* = 28 university students. They were in the same age range as the target group of the present study. These results were excluded from the main analysis.

### 5.3. Statistical Analysis

The software IBM SPSS version 26 was used for statistical analysis. Data description was presented as a percentage, corresponding to the relative number of participants answering a specific question. All of the questions corresponded to nominal or ordinal data. Adherence to the “golden rules” in question 16 (golden rules) was assessed using a metric, Likert-type scale (1–5): (1) “I never follow”, (2) “I usually follow”, (3) “Partly/partly”, (4) “I follow most of the time”, (5) “I always follow”. The option “it does not apply to me” was assigned with the value zero “0”, so that no influence is expected when calculating average values. The degree of compliance was estimated as average ± standard deviation. Significant differences by comparisons between groups “food-movements” vs. “no-participation” (control) were analysed using the Chi-square test. Differences between the groups were expressed as relative differences (%) between the average of compliance in each group. Negative percentages are indicative of a lower compliance of the food-movement group compared to the control. This was applied only to the rules with a statistically significant difference between both groups, based on the Chi-square test. The level of significance (*p*-values) was set to 0.05. As fixed factor was considered the participation in the food movements, namely the “food-movement” group versus the “no-participation” group. For graphical representation of the data, a bar diagram (mean values) was used; error bars correspond to standard deviations. 

## Figures and Tables

**Table 1:** Overview of EU regulated mycotoxins and their adverse effects, adapted from [1,17].

Mycotoxin	Regulated Foodstuffs	Reported Toxicities
Aflatoxins B1, B2, G1, G2 & M1	Cereals, grains and derived products, nuts, dried fruit, certain spices Milk and derived products, including infant formula (M1)	Hepatotoxic and carcinogenic in all species tested except mice Genotoxic: adducts formation in humans and animals
Ochratoxin A	Cereals, grains, dried vine fruit, Roasted coffee beans, ground roasted coffee, soluble coffee, wine, grape juice, certain spices	Nephrotoxic, teratogenic, immunosuppressant; rodent carcinogen
Patulin	Fruit juices, spirit drinks, solid apple products and apple juice, baby food	Causes damage in intestinal tissues, alterations in renal function
Deoxynivalenol	Cereals, grains and derived products such as pasta, bread, processed cereal-based foods and baby foods for infants and young children	Feed refusal and vomiting in domestic pigs Immunosuppression in mice
Zearalenone	Cereals, grains and derived products such as pasta, bread, processed cereal-based foods and baby foods for infants and young children	Estrogenic effects in pigs and experimental animals
Fumonisins B1 & B2	Maize and derived products	Leukoencephalomalacia in horses, pulmonary edema in pigs, neural tube defects in mouse embryos; rodent carcinogen
T-2 and HT-2	Unprocessed cereals and cereal products	Alimentary toxic aleukia, haemorrhage and vomiting
Citrinin	Food supplements based on rice fermented with red yeast *Monascus purpureus*	Nephrotoxic in pigs and rodents

**Table 2:** Seventeen golden rules: measures suggested by the BfR to reduce the risk of mycotoxin exposure in the household [33].

Rule	Description
1	Buy food as fresh as possible and consume it soon. Avoid hoarding purchases.
2	Store food properly (clean, dry) and in a cool place.
3	Clean bread boxes and similar items once a week and rinse with vinegar and water to prevent mould growth.
4	Remove bread crumbs from bread boxes as they favor mould growth.
5	Immediately dispose of food that is already mouldy, because mould is “contagious”.
6	The more liquid the food is, the faster the spread of mould and its toxins is likely to occur. Throw away such contaminated food.
7	Affected milk and milk products must no longer be consumed.
8	Mould-matured cheeses are harmless. To better distinguish it from “real” mould, cheese should always be stored in separate packaging.
9	Store cereals and flour in a cool, dry place and shake occasionally.
10	Buy fruit and vegetables that are as intact as possible, i.e., without injuries and bruises.
11	Mouldy jams and jellies should always be thrown away. Because of the lower sugar content, diet jams should always be stored in the refrigerator.
12	Rotten fruit should neither be eaten nor further processed into compote or jam.
13	If there are mouldy spots on bread, it should be thrown away whole.
14	In case of mould growth on meat and sausage, both should be discarded. In the case of air-dried sausage or ham, it is possible to cut this out generously and continue to consume the products.
15	Nuts that have become mouldy should be sorted out.
16	Spices should be bought in smaller quantities and consumed quickly.
17	Do not feed mouldy products to animals, as mycotoxins are just as harmful to them

**Table 3:** Risk perception of mycotoxins compared with other food xenobiotics. Values represent the % of participants.

	Xenobiotics			
Risk Perception	Pesticides (%)	Heavy Metals (%)	Microplastics (%)	Food Additives (%)
much less bad	4	7	8	4
somewhat less bad	24	43	34	18
equally bad	48	37	32	17
somewhat worse	21	12	21	40
much worse	3	1	5	21

## Data Availability

Additional information is in Supplementary Material.

## Supplementary Materials

The following are available online at https://www.mdpi.com/article/10.3390/toxins13110760/s1, Table S1. Question list and design included in the online survey.
