# Supplementary material for: Knowledge and Behavioral Habits to Reduce Mycotoxin Dietary Exposure at Household Level in a Cohort of German University Students
Source: Toxins (Basel). 2021 Oct 26;13(11):760. doi: 10.3390/toxins13110760 (PMC8618271; doi:10.3390/toxins13110760)
Supplement: Supplementary file 1 [file toxins-13-00760-s001.zip › toxins-1402665-supplementary.pdf]

toxins

MDPI

Supplementary Materials:

Knowledge and Behavioral Habits to Reduce Mycotoxin Dietary Exposure at Household Level in a Cohort of German University Students

Katherine Muñoz, Mara Wagner, Florian Pauli, Juliane Christ and Gerhard Reese

Table S1. Question list and design included in the online survey.

Description
1. How old are you?<br>o Younger than 20 years<br>o 20 to 29 years<br>o 30 to 39 years<br>o 40 to 49 years<br>o 50 to 59 years<br>o 60 years or older
2. Gender, please select one of the options<br>o female<br>o male<br>o diverse
3. How many people, including you, live in your household?<br>_______________
4. Educational level<br>o No qualification<br>o Special school leaving certificate<br>o Secondary school certificate<br>o Secondary school leaving certificate<br>o University degree<br>o Other: _______________
5. Monthly net income<br>This means the amount that is made up of your personal income (not that of the other household members) and is left over before the deduction of current expenses.<br>o less than 250<br>o 250€ up to € 499<br>o 500 € until 999 €<br>o 1000 € until 1499 €<br>o 1500 € until 1999 €<br>o 2000 € until 2999 €<br>o 3000 € until 3999 €

○ 4000 € till 4999 €<br>○ 5000 € and more<br>○ not specified
6. How much do you approximate spend on food each month?<br>This is only your personal expenditure, not that of the other household members. If you live together as a family, divide the total amount by the number of household members.<br>○ less than 50 €<br>○ 50 till 99 €<br>○ 100 till 199 €<br>○ 200 till 299 €<br>○ 300 till 399 €<br>○ 400 till 499 €<br>○ 500 € and more<br>○ Not specified
7. What is your current employment status?<br>Please select your main job.<br>○ In school<br>○ In apprenticeship<br>○ Studying<br>○ Employed<br>○ Civil servant<br>○ Self-employed<br>○ Working in the household<br>○ Unemployed / looking for work<br>○ Retired<br>○ Other: _______________
8. Which of the following statements applies most to you?<br>○ I mostly cook myself<br>○ Another member of my household usually does the cooking<br>○ I mostly eat in a canteen or restaurant<br>○ I usually get prepared food delivered (e.g. delivery service, "meals on wheels")<br>○ I mostly eat ready-made meals<br>○ Other: _______________
9. Which best describes your diet?<br>Please select the statement that applies I mostly cook myself.<br>○ I eat a vegan diet<br>○ I eat a vegetarian diet<br>○ I eat an omnivorous diet<br>○ The following applies to me: _______________
10. Which of the following statements apply to you?<br>Please select the statement that applies I mostly cook myself.<br>(For explanation: Foodsharing is an association that rescues food from the dustbin and distributes it, for example, through distributors called “FairDistributors”).<br>○ I regularly eat food from foodsharing<br>○ I eat food from dumpster diving<br>○ I eat mainly organic food

- ○ The following applies to me: _______________
- ○ None of this applies to me.

11. We have compiled statements on the topic of nutrition. Please rate to what extent you agree or disagree with each statement.

	Do not agree at all	Strongly disagree	Partly/ partly	Strongly agree	Fully agree
Nutrition is an important topic for me.	○	○	○	○	○
I think that I am very well versed in nutritional issues.	○	○	○	○	○
I pay a lot of attention to the quality of my food.	○	○	○	○	○
The price of food is more important to me than the quality.	○	○	○	○	○

12. How often do you eat or use...

	Daily	3-6 times a week	1-2 times a week	1-3 times a month	Rarely or never
Nuts and shell fruits (e.g. hazelnuts, peanuts, almonds, pistachios)	○	○	○	○	○
Oil seeds (e.g. linseed, pumpkin seeds, sesame seeds)	○	○	○	○	○
Dried fruits	○	○	○	○	○
Cow's milk	○	○	○	○	○
Cereals, bread and other bakery products	○	○	○	○	○
Breakfast cereals (e.g. cornflakes, oatmeal)	○	○	○	○	○
Instant coffee	○	○	○	○	○
Apple juice / grape juice	○	○	○	○	○
Dried spices (e.g. chilli, paprika, nutmeg, curcuma)	○	○	○	○	○

13. Do you know what mould toxins are?
- ○ Yes
- ○ No

14. Have you ever heard the term "mycotoxins"?
- ○ Yes
- ○ No

15. Have you ever heard of "ergot poisoning"?
   - ○ Yes
   - ○ No

Intervention text

Here is a brief information about mycotoxins:

Mycotoxins are toxic compounds produced naturally by various species of fungi.

Mycotoxins enter the food chain as a result of infection of crops before or after harvest and typically occur in foods such as cereals, dried fruits, nuts and spices. The presence of mycotoxins in food and feed can cause adverse health effects in humans and animals, ranging from gastrointestinal and kidney diseases to immune deficiency and cancer.

Exposure to mycotoxins can occur through the consumption of contaminated food or through animals fed contaminated feed. The most common mycotoxins that pose a risk to human and animal health include aflatoxins, ochratoxin A and Fusarium toxins such as deoxynivalenol.

As temperature and humidity are important parameters for fungal growth, climate change is expected to affect the occurrence of mycotoxins.

Golden rules

16. Below you will find some recommendations for reducing mycotoxin contamination in food. Please tick in each case whether you currently follow this recommendation or not.

	I never follow	I usually do not follow	Partly/ partly	I follow most of the time	I always follow	Does not apply to me
(R_01) Buy food as fresh as possible and consume it soon. Avoid hoarding.	○	○	○	○	○	○
(R_02) Store food properly (clean, dry) and in a cool place.	○	○	○	○	○	○
(R_03) Clean bread boxes and similar items once a week and wipe with vinegar to prevent mould growth.	○	○	○	○	○	○
(R_04) Remove bread crumbs from bread boxes as they promote mould growth.	○	○	○	○	○	○
(R_05) Immediately dispose of food that is already mouldy, as mould is "contagious".	○	○	○	○	○	○
(R_06) The more liquid the food is, the faster the mould and its toxins can spread. Throw away such infested food.	○	○	○	○	○	○
(R_07) Infested milk and dairy products must no longer be consumed.	○	○	○	○	○	○
(R_08) Mould- matured cheeses are harmless. To better distinguish it from	○	○	○	○	○	○

"real" mould, cheese should always be stored in separate packaging.						
(R_09) Store cereals and flour in a cool, dry place and shake occasionally.	○	○	○	○	○	○
(R_10) Buy fruit and vegetables that are as intact as possible, i.e. without injuries or bruises.	○	○	○	○	○	○
(R_11) Mouldy jams and jellies should always be thrown away. Because of the lower sugar content, diet jams should always be stored in the refrigerator.	○	○	○	○	○	○
(R_12) Rotten fruit should neither be eaten nor processed further into compote or jam.	○	○	○	○	○	○
(R_13) If there is mould on bread, it should be thrown away whole.	○	○	○	○	○	○
(R_14) If meat or sausage is mouldy, both should be discarded. In the case of air-dried sausage or ham, it is possible to cut this out generously and continue to eat the products.	○	○	○	○	○	○
(R_15) Individual mouldy nuts should be discarded.	○	○	○	○	○	○
(R_16) Spices should be bought in smaller quantities and consumed quickly.	○	○	○	○	○	○
(R_17) Do not feed mouldy products to animals, as mycotoxins are just as harmful to them.	○	○	○	○	○	○

17. In the following we have again compiled some statements. Please indicate to what extent you personally agree or disagree with these statements.

	Do not agree at all	Strongly disagree	Partly/ partly	Strongly agree	Fully agree
I try to avoid health problems even before I notice symptoms.	○	○	○	○	○
I think a lot about health risks and try to prevent them.	○	○	○	○	○

I try to protect myself from health risks I hear about.	○	○	○	○	○
Health risks only worry me when they affect me or someone close to me. Nowadays there are so many things that can harm you. I don't worry about that.	○	○	○	○	○
I am often worried about health risks I have heard about, but I don't do anything about them.	○	○	○	○	○
I only do something about health risks I have heard about when I have a problem with them myself.	○	○	○	○	○

18. How do you rate the following statements on mycotoxins? For each statement, please mark with a cross the extent to which you agree or disagree with it.

	Do not agree at all	Strongly disagree	Partly/ partly	Strongly agree	Fully agree
I was already aware of the health hazards of mould toxins in food.	○	○	○	○	○
For me personally, I consider the health hazards of mycotoxins to be insignificant.	○	○	○	○	○
The described health hazards of mycotoxin exposure frighten me.	○	○	○	○	○
I find the health risk from mycotoxins particularly alarming because the adverse health effects only appear after many years.	○	○	○	○	○
I consider the health risk from mycotoxins to be insignificant for the general public in Germany.	○	○	○	○	○
Mycotoxins are a great risk for young children.	○	○	○	○	○
Mycotoxins have the potential to make very many people ill at once.	○	○	○	○	○
Mycotoxins are a serious threat to health.	○	○	○	○	○
As a consumer, I have no way to protect myself from mycotoxin exposure.	○	○	○	○	○

19. How high do you rate your overall ability to influence the prevention of mycotoxins in your food?

- Very high
- Rather high
- Partly/Partly
- Rather low
- Very low

20. To what extent do you think you can protect your health by avoiding mycotoxins in your food?

- Very strongly
- Rather strongly
- Partly/Partly
- Rather low
- Very low

17. How do you assess the health risk posed by mycotoxins compared to other risks in food?

I rate the health risk from mycotoxins...	Much less bad	Somewhat less bad	Equally bad	Somewhat worse	Much worse
compared to the risk from pesticide residues as...	○	○	○	○	○
compared to the risk from heavy metals as...	○	○	○	○	○
compared to the risk from microplastics as...	○	○	○	○	○
compared to the risk from food additives as...	○	○	○	○	○

22. Where do you most frequently obtain information on health-related topics?
Please select your most frequent source of information.

- Social media
- Through friends / acquaintances
- From health professionals (doctors/pharmacists)
- Public online information pages and institutes, e.g. the Federal Institute for Risk Assessment
- Other: _______________

23. How would you rate the health risks of mycotoxins in general?

- Not at all risky
- Rather not risky
- Partly/Partly
- Rather risky
- Extremely risky
